# Acid Tolerant and Acidophilic Microalgae: An Underexplored World of Biotechnological Opportunities

**DOI:** 10.3389/fmicb.2022.820907

**Published:** 2022-01-27

**Authors:** Fabian Abiusi, Egbert Trompetter, Antonino Pollio, Rene H. Wijffels, Marcel Janssen

**Affiliations:** ^1^Bioprocess Engineering, AlgaePARC, Wageningen University and Research, Wageningen, Netherlands; ^2^Department of Biology, University of Naples Federico II, Naples, Italy; ^3^Faculty of Biosciences and Aquaculture, Nord University, Bodø, Norway

**Keywords:** extremophilic microalgae, biomass productivity, mixotrophy, biomass yield on substrate, temperature optima, *Galdieria*, *Chlamydomonas acidophila*, *Stichococcus bacillaris*

## Abstract

Despite their large number and diversity, microalgae from only four genera are currently cultivated at large-scale. Three of those share common characteristics: they are cultivated mainly autotrophically and are extremophiles or tolerate “extreme conditions.” Extreme growth conditions aid in preventing contamination and predation of microalgae, therefore facilitating outdoor cultivation. In search for new extremophilic algae suitable for large-scale production, we investigated six microalgal strains able to grow at pH below 3 and belonging to four genera; *Stichococcus bacillaris* ACUF158, *Chlamydomonas acidophila* SAG 2045, and *Chlamydomonas pitschmannii* ACUF238, *Viridiella fridericiana* ACUF035 and *Galdieria sulphuraria* ACUF064 and ACUF074. All strains were cultivated autotrophically at light intensity of 100 and 300 μmol m^−2^ s^−1^ and pH between 1.9 and 2.9. The autotrophic biomass productivities were compared with one of the most productive microalgae, *Chlorella sorokiniana* SAG 211-8K, grown at pH 6.8. The acid tolerant strains have their autotrophic biomass productivities reported for the first time. Mixotrophic and heterotrophic properties were investigated when possible. Five of the tested strains displayed autotrophic biomass productivities 10–39% lower than *Chlorella sorokiniana* but comparable with other commercially relevant neutrophilic microalgae, indicating the potential of these microalgae for autotrophic biomass production under acidic growth conditions. Two acid tolerant species, *S. bacillaris* and *C. acidophila* were able to grow mixotrophically with glucose. *Chlamydomonas acidophila* and the two Galdieria strains were also cultivated heterotrophically with glucose at various temperatures. *Chlamydomonas acidophila* failed to grow at 37°C, while *G. sulphuraria* ACUF64 showed a temperature optimum of 37°C and *G. sulphuraria* ACUF74 of 42°C. For each strain, the biomass yield on glucose decreased when cultivated above their optimal temperature. The possible biotechnological applications of our findings will be addressed.

## Introduction

Microalgae are a diverse, polyphyletic group of organisms, boasting estimated species number between 2,00,000 and several million (Norton et al., 1996). In addition to cultivating microalgae for food and feed they also hold promise for a plethora of new products and applications. Microalgae are commonly grown exploiting their photoautotrophic capacity (henceforth referred to as autotrophic), in which cells harvest light energy, use carbon dioxide (CO_2_) as a carbon source, and release oxygen (O_2_) as a byproduct. Alternatives to autotrophic cultures are chemo-organotrophic (henceforth referred to as heterotrophic) cultures in which organic carbon, such as sugars and organic acids, are used as carbon sources in the absence of light. Despite the enormous diversity, only four genera of microalgae are cultivated at large-scale: *Arthrospira* (Spirulina), *Chlorella*, *Dunaliella*, and *Haematococcus* (Pulz and Gross, 2004). Excluding *Chlorella*, which is mainly produced heterotrophically in fermenters utilizing glucose or acetic acid (Iwamoto, 2004), the other three genera mentioned are cultivated mainly autotrophically and are extremophiles or tolerate “extreme conditions.”

Extreme conditions are, for example, unusually high or low temperature, high or low pH, or high osmotic pressure. If a strain has at least one of its growth optima falling into such a range, it is considered an extremophile, and if it has more than one optimum in such categories, the term is “poly-extremophile.” *Spirulina* is cultivated in alkaline media (pH 8.5–10.5), *Dunaliella* at high NaCl concentrations (35–300 g/L; Oren, 2014) and also *Haematococcus* can tolerate high salinity, which is used to promote accumulation of the red pigment astaxanthin within the cells (Oslan et al., 2021). Extreme growth conditions aid in preventing contamination and predation of microalgae, therefore facilitating their outdoor cultivation.

Bacterial contamination is a notable challenge when microalgae are cultivated in a medium containing organic carbon, as microalgae have a growth rate one order of magnitude lower than their competitors. In order to avoid bacterial contaminations, heterotrophic cultivation of microalgae is performed in bioreactors, where all inputs (liquid media or gasses) are sterilized and the process is optimized to operate axenically. With some microalgal species, autotrophic and heterotrophic cultivation can be combined in so-called “mixotrophic” cultivation. In this trophic mode, light and organic carbon are simultaneously provided and both heterotrophic and autotrophic metabolisms operate concurrently within a single microalgal monoculture. Mixotrophic cultivation can significantly increase biomass productivity and concentration, while utilizing light energy with the same photosynthetic efficiency of an autotrophic culture (Abiusi et al., 2020a,b).

When sunlight is used as a light source, mixotrophic cultivation of microalgae is performed in photobioreactors, characterized by high surface/volume ratio to maximize the light supply rate (Posten, 2009). Although it might be technically feasible to operate closed sunlit photobioreactors without bacterial contamination, maintaining axenic conditions in an outdoor photobioreactor, especially when a medium contains a source of organic carbon is challenging. In a pursuit of a strategy to prevent bacterial contamination under mixotrophic conditions, Henkanatte-Gedera et al. (2017) demonstrated that lowering pH of unsterilized urban primary effluent to a value of 2 resulted in a complete removal of pathogens and reduced the initial bacterial population by 98%, allowing an acidophilic microalgae to be the primary organism growing in such a nutrient-rich medium. Contamination by unwanted microorganisms is not only a challenge in the presence of an organic substrate. There is a wide diversity of protist taxa (e.g., amoeba, flagellates, and ciliates) able to graze on microalgae, threatening the commercial success of the developing microalgal industry (Day et al., 2017). Several studies demonstrate that pH affects microbial community diversity more than any other parameter tested (Merino et al., 2019). Moreover, it is well documented that few ciliate and rotifer can survive pH below 3 (Seckbach, 2007).

During the last two decades, increasing attention has been paid toward acidophilic and acid tolerant microalgae and their possible biotechnological applications such as the production of acid stable pigments (Rahman et al., 2017; Ferraro et al., 2020a), rare earth metal biorecovery (Minoda et al., 2015), extremozymes (Rahman et al., 2020) and extremolytes (Martinez-Garcia and van der Maarel, 2016). Despite their potential, most of the research on acidophilic microalgae has been focused on one species only: *Galdieria sulphuraria* (Sydney et al., 2019).

The aim of this study was to find microalgal strains able to grow at low pH sufficiently to exhibit potential for large-scale production with or without utilizing organic carbon. In order to achieve this goal, we investigated the autotrophic biomass productivities of six acid tolerant microalgal strains at pH below 3 and compared their productivity with the neutrophilic *Chlorella sorokiniana*. Three strains were chosen since there were previous reports on their ability to use glucose, namely: *Stichococcus bacillaris* ACUF158, *Chlamydomonas acidophila* SAG 2045, and *Chlamydomonas pitschmannii* ACUF238 (Martinez et al., 1987; Pollio et al., 2005; Spijkerman, 2007). *Viridiella fridericiana* ACUF035 was suggested from the Algal Collection of University Federico II of Naples (ACUF) due to good autotrophic performance. Two strains of *G. sulphuraria*, the most studied acidophilic microalgae species, were compared with the acid tolerant strains. *Galdieria sulphuraria* ACUF064 and ACUF074 were selected based on the results of a screening of 42 *Galdieria* strains (Graziani et al., 2013) as they displayed the best autotrophic and heterotrophic growth rates. The acid tolerant microalgae strains performing well autotrophically were then also cultivated mixotrophically and, when possible, heterotrophically.

## Materials and Methods

### Organism, Media, and Cultivation Conditions

Seven microalgal strains were used in this study. *Stichococcus bacillaris* ACUF158, *C. pitschmannii* ACUF238, *V. fridericiana* ACUF035, *G. sulphuraria* ACUF064, and *G. sulphuraria* ACUF074 were obtained from the Algal Collection of University Federico II of Naples (ACUF). *Chlamydomonas acidophila* SAG 2045 and *C. sorokiniana* SAG 211-8K were obtained from the algae culture collection of Göttingen University (SAG). For each strain maintenance flasks were prepared. Maintenance cultures were cultivated autotrophically by placing 250 ml flasks in an incubator with orbital shaker set at 100 rpm. Incubator headspace was enriched with 2% v/v CO_2_ and the flasks were illuminated 24/24 from the top by fluorescent lamps at a photon flux density (PFD) of 100 μmol m^−2^ s^−1^.

*Chlorella sorokiniana* SAG 211-8K was cultivated at pH 6.8 ± 0.1 at 37°C in M8a medium (Abiusi et al., 2020a) with ammonium chlorine as a nitrogen source. All the other strains were cultivated initially at pH 2.1 ± 0.2 adjusting the pH with 2 M H_2_SO_4_. The medium was prepared according to Abiusi et al. (2021) composed of the following salts (in mol L^−1^): 2.2 10^−3^ KH_2_PO_4_, 20.0 10^−3^ (NH_4_)_2_SO_4_, 1.6 10^−3^ MgSO_4_ 7 H_2_O, 0.1 10^−3^ CaCl_2_, 0.16 10^−3^ EDTA ferric sodium salt, 0.05 10^−3^ Na_2_EDTA 2H_2_O, 0.9 10^−3^ NaCl, 0.2 10^−3^ H_3_BO_3_, 20.2 10^−6^ MnCl_2_ 4H_2_O, 20.6 10^−6^ ZnCl_2_, 8.0 10^−6^ CuSO_4_ 5H_2_O, 4.1 10^−6^ Na_2_MoO_4_ 2H_2_O, and 4.2 10^−6^CoCl_2_ 6H_2_O. If no growth was observed, pH was increased to 2.9 ± 0.2. The two *Galdieria* strains were maintained at 37°C, while the other acid tolerant strains were placed in two different incubators, set at 25 and 37°C.

### Autotrophic Flasks Experiment

In this study, we assessed the autotrophic performance of four acid tolerant microalgal strains, with a pH optimum above 3 but able to grow at pH 3 or lower, namely: *S. bacillaris* ACUF158, *C. acidophila* SAG 2045, *C. pitschmannii* ACUF238, *V. fridericiana* ACUF035, and two acidophilic strains *G. sulphuraria* ACUF064 and *G. sulphuraria* ACUF074, with a pH optimum below 3.

**Figure 1:**
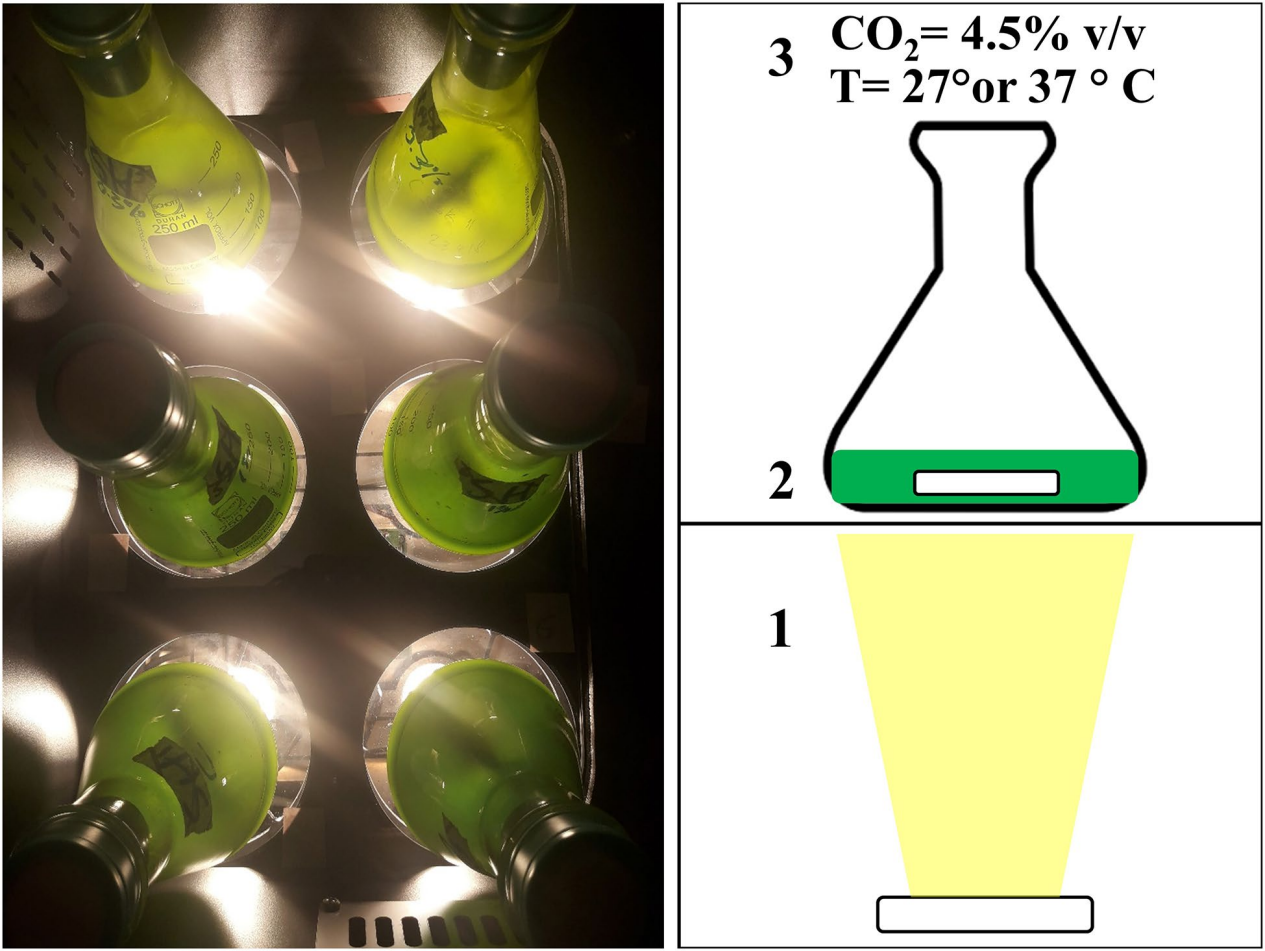
Picture (left) and schematic view of the incubator used during the autotrophic experiment. Light was provided from below using a LED (1), flasks were stirred at 100 rpm with a magnetic rod (2), and the head space of the incubator was enriched with 4.5% v/v carbon dioxide (CO_2_; 3). Temperature of the incubator was maintained constant, either at 27°C or at 37°C, depending on the experiment (3).

The autotrophic growth of these six microalgal strains was studied in 250 ml flasks filled with 100 ml of culture in a home-made incubator described by Abiusi et al. (2020a) and depicted in Figure 1. In this incubator illumination was provided 24/24 from below using warm-white LED at a PFD of 100 ± 15 and 300 ± 25 μmol m^−2^ s^−1^. The position of each flask in the incubator was changed daily to minimize differences in light intensity within the incubator. Flasks were stirred at 100 rpm with a magnetic rod and the headspace of the incubator was enriched with 4.5% v/v CO_2_.

The four acid tolerant strains were cultivated at a temperature and pH in which growth was observed during the pre-cultivation summarized in Table 1. The two acidophilic *G. sulphuraria* strains were cultivated at pH 1.8 ± 0.2. In each experiment, pH was measured at the beginning and at the end of the experiment. The neutrophilic strain *C. sorokiniana* SAG 211-8K and the two *G. sulphuraria* strains were cultivated at 37°C.

**Table 1:** Temperature and pH used during the autotrophic experiment.

Strain	pH optimum	pH	T (C°)
*Chlorella sorokiniana* SAG 211-8K	Neutrophilic	6.8 ± 0.1	37
*Galdieria sulphuraria* ACUF064	Acidophilic	1.9 ± 0.2	37
*Galdieria sulphuraria* ACUF074	Acidophilic	2.0 ± 0.2	37
*Viridiella fridericiana* ACUF035	Acid tolerant	2.1 ± 0.2	27
*Chlamydomonas acidophila* SAG 2045	Acid tolerant	2.2 ± 0.1	27
*Stichococcus bacillaris* ACUF158	Acid tolerant	2.9 ± 0.3	27
*Chlamydomonas pitschmannii* ACUF238	Acid tolerant	2.8 ± 0.2	27

The pH was measured at the beginning and at the end of the experiment and is reported with the SD of the two measurements.

Each experiment was conducted in duplicate. Maintenance flasks were used as inoculum. Cultures were pre-cultivated using the maintenance flasks as inoculum and cultures were adapted to the respective light and temperature settings for at least 1 week. Provided that linear growth was observed during this adaptation time, the pre-culture was used as an inoculum for the actual autotrophic experiment starting at an optical density 1.2 ± 0.3 at 100 μmol m^−2^ s^−1^ and 4.5 ± 1.2 at 300 μmol m^−2^ s^−1^ measured at 750 nm (*OD_750_*).

During the batch, one or two samples per day were taken from each flask to measure *OD_750_* and the photosystem II maximum quantum yield of photochemistry (*QY* or Fv/Fm). The microalgal concentration was assessed by converting *OD_750_* into biomass concentration (*C_x_* g_x_ L^−1^), using *OD_750_*/*C_x_* linear correlation pre-determined for each strain and light intensity. The validity of this correlation was confirmed at the end of each experiment by an additional measurement of dry weight and optical density. The volumetric biomass production rate *r_x_* (g_x_ L^−1^ day^−1^) was calculated from a linear regression of the increase of biomass concentration (*C_x_*) over time during the batch.

### Mixotrophic Microplate Experiment

The ability of *S. bacillaris* ACUF158, *C. acidophila* SAG 2045, and *V. fridericiana* ACUF035 to grow mixotrophically utilizing glycerol, acetic acid, and glucose under 24/24 lighting was tested in 24-well microplates at pH 2.9 ± 0.2 (Costar 3524, Corning, United States). Glucose, acetic acid, and glycerol were supplemented to the autotrophic medium based on their carbon content. In order to provide 1 g L^−1^ of organic carbon, 2.75 g L^−1^, 2.50 g L^−1^, and 2.56 g L^−1^ of glucose monohydrate, acetic acid, and glycerol were used. The mixotrophic performance was evaluated using an autotrophic culture grown under the same conditions in a medium without an organic carbon source as a reference.

Maintenance cultures were diluted to an *OD_750_* of 0.3–0.6 with the specific media required and each well was filled with 1 ml of this culture. Each medium was tested in triplicate. The 24-well plates were placed in an incubator at 25°C, headspace was enriched with 2% v/v CO_2_ and shaken at 100 rpm (orbital shaker). Light was provided from the top by fluorescent lamps (Sylvania *CF*-LE 55 W) giving a light intensity of 75 μmol m^−2^ s^−1^. Microalgal growth was followed by measuring *OD_680_* and *OD_750_* using a plate reader (Infinite Nanoquant M200, Tecan, Switzerland). The ratio *OD_680_*/*OD_750_* was used as an indicator for the amount of chlorophyll per unit biomass and therefore of possible bacterial contaminations.

### Heterotrophic Flasks Experiment

*Chlamydomonas acidophila* SAG 2045, *G. sulphuraria* ACUF064, and *G. sulphuraria* ACUF074, where cultivated heterotrophically under the same conditions used for the autotrophic experiment but supplementing the medium with 2.75 g L^−1^ glucose monohydrate without illumination. The experiment was conducted using the same procedure described by Abiusi et al. (2021). Several temperatures were tested. At each temperature cultures were adapted to heterotrophic growth for at least 2 weeks. Specific growth rate (*μ*) was calculated by plotting the natural logarithm of *OD_750_* over time of cultures growing exponentially.

During the experiments, multiple samples were taken per day until glucose was depleted. The concentration of microalgae was assessed by measuring optical density at 750 nm (*OD_750_*). A 1 ml aliquot of sample was centrifuged to obtain a clear supernatant for the glucose concentration measurement. The microalgae concentration was assessed by converting *OD_750_* into *C_x_* using a OD_750_/*C_x_* linear correlation pre-determined for each strain and temperature. The validity of this correlation was confirmed at the end of each experiment by an additional measurement of dry weight and optical density.

The heterotrophic biomass yield on substrate (*Y_x/s_*, g_x_ g_s_^−1^) was calculated using the following equation:


$$Y_{x/s}=\frac{C_{xe}-C_{x0}}{S_{0}-S_{e}}$$

where *C_x0_*/*C_xe_* and *S_0_*/*S_e_* are the biomass and the glucose concentrations (g L^−1^) respectively at the start and the end of the exponential phase.

### Analytical Methods

The PFD (μmol m^−2^ s^−1^) was measured with a LI-COR 190-SA 2π PAR quantum sensor and dry weight concentration (*C_x_*, g_x_ L^−1^), optical density at 750 nm (*OD_750_*), and the photosystem II maximum quantum yield of photochemistry (*QY* or Fv/Fm) were determined according to Abiusi et al. (2020a). The presence of possible microbial contaminants was assessed both by optical and fluorescent microscopy after staining the sample with SYBER green I (Sigma-Aldrich, United States) according to Abiusi et al. (2020a).

Samples were taken aseptically from flasks one or two times per day. In the heterotrophic experiment, aliquots of 1 ml were centrifuged at 20,000 rpm (10 min). The supernatant was immediately analyzed for glucose content. Glucose concentration was determined using a bioanalyzer (YSI 2700, YSI Life Sciences, United States) that couples an enzymatic reaction of glucose with electrochemical detection.

### Statistical Analysis

Autotrophic and heterotrophic experiments were conducted in biological duplicate (*n* = 2), while mixotrophic experiment in biological triplicate (*n* = 3). Figures reported the SD of these replicates. Significant difference between strains cultivated in flasks under autotrophic and heterotrophic condition or between autotrophic and mixotrophic culture in microplate were analyzed by one-way ANOVA. The significance level was *p* < 0.05.

## Results

### Autotrophic Growth

Four acid tolerant and two acidophilic microalgae strains were tested for their autotrophic performance at two light intensities (100 and 300 μmol m^−2^ s^−1^) and compared to the neutrophilic *C. sorokiniana* SAG 211-8K. Maintenance cultures were prepared before initiating the experiment. These were kept at a pH 2.0 ± 0.2 at 37°C. For the acid tolerant strains additional maintenance cultures were kept at 25°C. At pH 2.0 ± 0.2 no growth was obtained in *C. pitschmannii* ACUF238 and *S. bacillaris* ACUF158 cultures regardless of the temperature applied. For this reason, the pH was increased to 2.9 ± 0.2. After the increase of pH those two strains succeeded in growing at 25°C but not at 37°C. *Viridiella fridericiana* ACUF035 and *C. acidophila* SAG 2045 successfully grew at pH 2.1 ± 0.2 but no growth was observed at 37°C. The presence in the medium of two buffers, by H_3_PO_4_ ⇌ H_2_PO_4_^−^ + H^+^ (pKa_1_ = 2.14) and H_2_SO_4_ + H_2_O ⇌ H^+^ + HSO^−^_4_ (pK_a1_ = 1.92), maintained the pH stable across the experiments. Table 1 summarizes the final temperatures and pH used during the autotrophic experiment at two light intensities.

**Figure 2:**
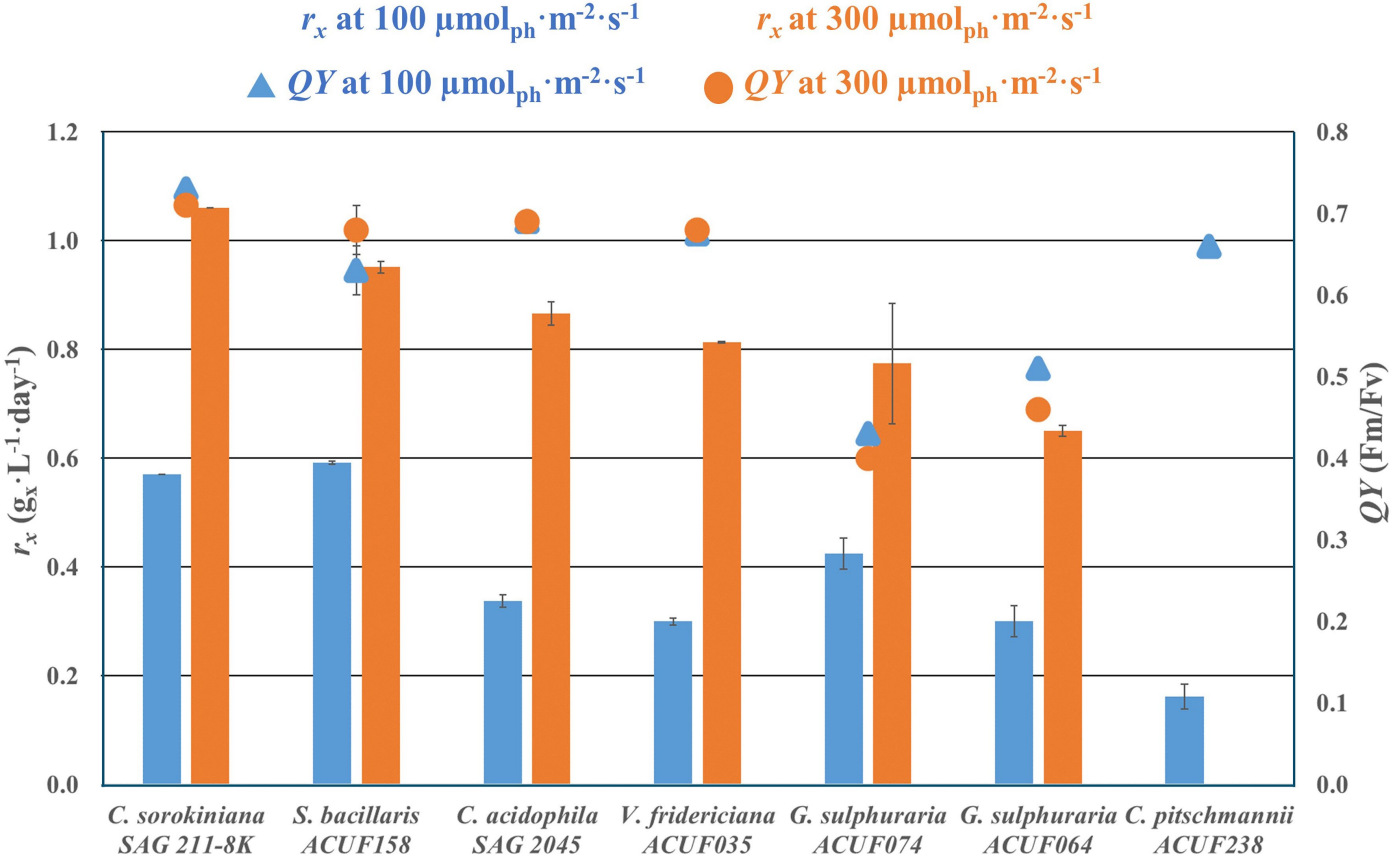
Volumetric biomass production rate *r_x_* (g_x_ L^−1^ day^−1^) and photosystem II maximum quantum yield of photochemistry (*QY*, F_v_/F_m_) of autotrophic culture grown at 100 and 300 μmol m^−2^ s^−1^. The data are presented as average of the biological duplicate (*n* = 2) and reported with the SD of the measurements.

All of the tested strains succeeded in growing at 100 μmol m^−2^ s^−1^ (Figure 2; Supplementary Figures 1–7). No significant difference (*p* < 0.05) was found between the biomass productivities of *S. bacillaris* and *C. sorokiniana* (0.6 g L^−1^ day^−1^), while the other five strains had a biomass productivity 25–72% lower than *C. sorokiniana*. When the strains were cultivated at 300 μmol m^−2^ s^−1^, *C. pitschmannii* did not grow and *C. sorokiniana* expressed the highest productivity (1.1 g L^−1^ day^−1^). The other five strains showed a productivity which was 10–39% lower than *C. sorokiniana*. Given the poor autotrophic performance of *C. pitschmannii*, this strain was discontinued from further investigations.

The photosystem II maximum quantum yields of photochemistry were between 0.66 and 0.73 in *C. sorokiniana* and the acid tolerant strains with no significantly differences (*p* < 0.05) between 100 and 300 μmol m^−2^ s^−1^. At 100 μmol m^−2^ s^−1^, *G. sulphuraria* ACUF064 and *G. sulphuraria* ACUF074 had *QY* 30% lower than other strains. The *QY* of both strains significantly decreased (*p* > 0.05) further at 300 μmol m^−2^ s^−1^, resulting in a *QY* of 0.46 ± 0.1 for *G. sulphuraria* ACUF064 and of 0.40 ± 0.1 for *G. sulphuraria* ACUF074.

### Mixotrophic Growth

**Figure 3:**
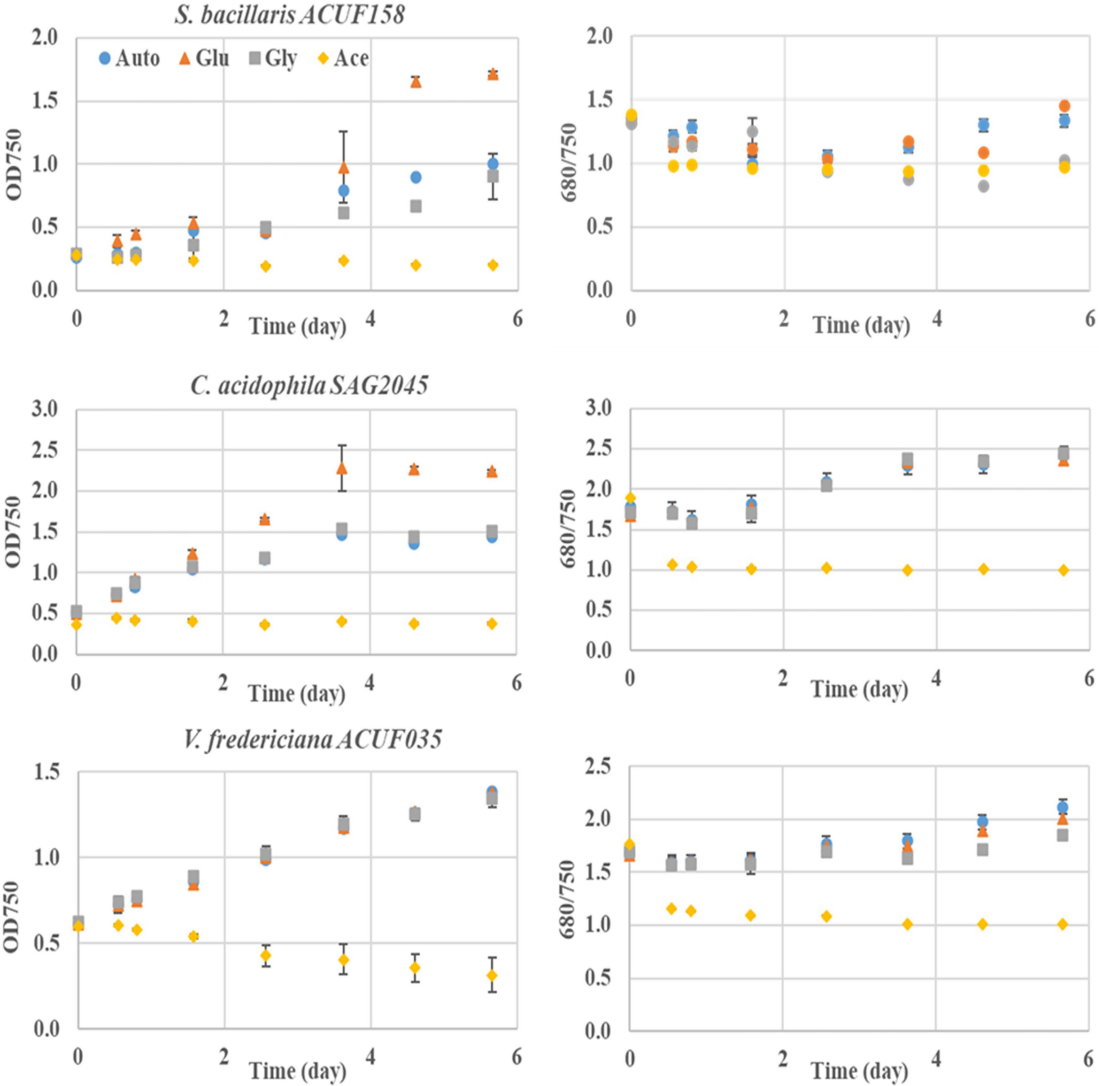
Mixotrophic performance in the presence of 1 g/L of individual carbon source such as glucose (triangle), glycerol (square), and acetic acid (diamond) of three acid tolerant microalgal cultivated at pH 2.9 ± 0.2 strains under 24/24 lighting. An autotrophic culture (dot) is used as reference.

The ability of *S. bacillaris* ACUF158, *C. acidophila* SAG 2045, and *V. fridericiana* ACUF35 to utilize glucose, acetic acid, and glycerol in presence of light was tested on 24-well microplates (Figure 3). The final *OD_750_* reached for *S. bacillaris* ACUF158 and *C. acidophila* SAG 2045 grown in presence of glucose was significantly higher than the autotrophic cultures grown under similar conditions. In both strains *OD_680_*/*OD_750_* remained constant suggesting that the cultures were axenic and that glucose boosted algal growth. Microscopic observation from samples taken at the end of the experiment confirmed axenicity (Supplementary Figures 8–10). *Viridiella fridericiana* ACUF35 was growing similarly in the presence of glucose as the corresponding autotrophic culture indicating that the culture was not able to utilize this substrate. Glycerol did not have any effect on the three tested strains, while acetic acid was lethal for all of them.

### Heterotrophic Growth

**Figure 4:**
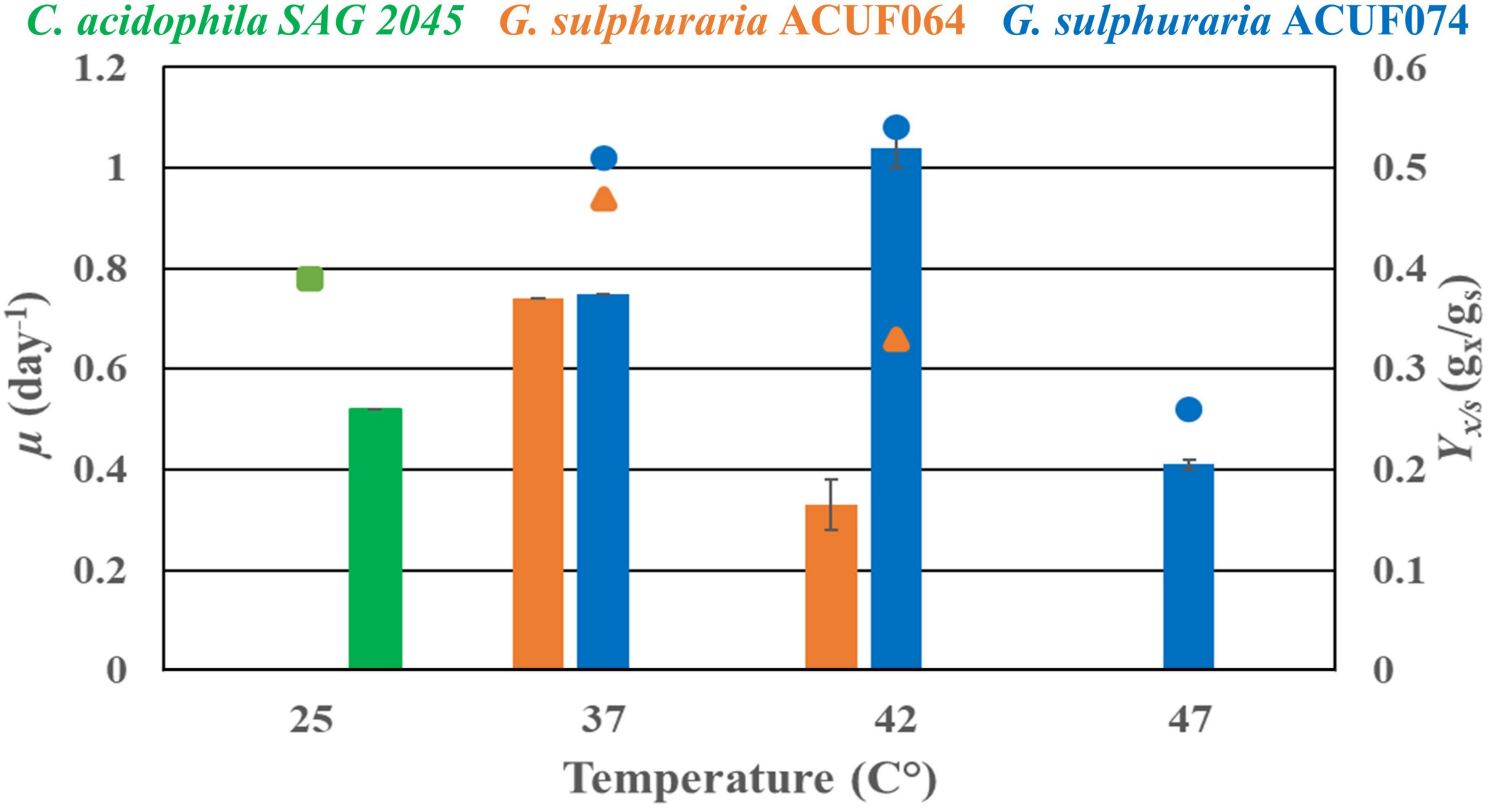
Heterotrophic specific growth rate (*μ*) and biomass yield substrate (*Y_x/s_*) of *Chlamydomonas acidophila* SAG 2045 (green), *Galdieria sulphuraria* ACUF064 (orange), and *Galdieria sulphuraria* ACUF074 (blue) grown on glucose at different temperatures.

Heterotrophic experiments were conducted with *C. acidophila* SAG 2045, *G. sulphuraria* ACUF064, and *G. sulphuraria* ACUF074 to determine specific growth rate (*μ*) and biomass yield on substrate (*Y_x/s_*) using glucose. Cultures were adapted for 2 weeks before starting the experiment under heterotrophic conditions. During this adaptation period, the cultures were diluted regularly with fresh medium to maintain exponential growth. During the adaptation time, all the cultures lost their pigmentation and became pale. *Chlamydomonas acidophila* cultivated at 25°C had a specific growth rate of 0.52 day^−1^ and a biomass yield on substrate of 0.4 g_x_ g_s_^−1^, while no growth was observed at 37°C (Figure 4).

The two *G. sulphuraria* strains grown at 37°C had similar specific growth rates and biomass yields on substrate, 0.75 day^−1^ and 0.5 g_x_ g_s_^−1^ (Figure 4). When temperature was increased to 42°C the specific growth rate of *G. sulphuraria* ACUF064 decreased to 0.33 day^−1^, while the specific grow rate of *G. sulphuraria* ACUF074 increased to 1.04 day^−1^ to decline again to 0.41 day^−1^ at 47°C. For each strain, the biomass yield on glucose decreased as soon as it was cultivated above its optimal temperature.

## Discussion

In the present study six microalgal strains, belonging to four different microalgal genera, were cultivated autotrophically at a pH below 3, under similar conditions. The autotrophic performances of those six microalgal strains were compared with neutrophilic *C. sorokiniana* SAG 211-8K, which is one of the fastest growing microalgae (Cuaresma et al., 2009). Five of the tested strains showed an autotrophic biomass productivity which was 10–39% lower than *C. sorokiniana* but comparable with other commercially relevant neutrophilic microalgae such as *Isocrysis lutea* (Gao et al., 2020), *Rhodomonas* sp. (Oostlander et al., 2020), and *Nannochloropsis* sp. (Benvenuti et al., 2016) indicating the potential of these microalgae for autotrophic biomass production under acidic growing conditions.

Literature concerning autotrophic productivity of acid tolerant microalgae grown below pH 3 is limited. Most studies on acid tolerant microalgae isolated from acid waters are not focusing on biomass production but were conducted from taxonomic (Albertano et al., 1991, 2000), evolutionary (Costas et al., 2007), or ecological viewpoints (Aguilera et al., 2007). In the present work, autotrophic biomass productivities of the studied acid tolerant strains are reported for the first time. One of the few previous works on biomass productivity of an acid tolerant microalgae was conducted by Cuaresma et al. (2011) in a 1 L photobioreactor. The authors cultivated a newly isolated acid tolerant *C. acidophila* strain at pH 2.5 obtaining a biomass areal productivity of 20 g m^−2^ day^−1^. The acid tolerant strains used in our study displayed a biomass areal productivity of 15–18 g m^−2^ day^−1^ when cultivated at 300 μmol m^−2^ s^−1^. Those areal productivities were derived from the volumetric productivity considering the bottom of the flask as illuminated surface (Figure 1). The resulting areal productivities obtained in flasks with the two *G. sulphuraria* strains are comparable to the productivities observed in our two recent studies in a 2 L photobioreactor (Abiusi et al., 2021, 2022). The data obtained from our flask incubator (Figure 1) therefore represents cultivation in a bench-scale photobioreactor. This is due to the specific setup of our experiment where algae, grown in flasks at a controlled temperature and elevated CO_2_, are illuminated from below which leads to a high surface/volume ratio (50.3 m^−1^) and a high light supply rate. The high light regime can also explain why linear growth was maintained until a high biomass concentration of 9 g L^−1^ was reached (Supplementary Figures 1–6).

Other studies on acid tolerant strains often report only the autotrophic specific growth rate (*μ*) measured in the exponential growth phase (Gerloff-Elias et al., 2005; Olivieri et al., 2011). Although, the specific growth rate is not sufficient to predict biomass productivity, it may be used to compare different conditions and to give a first indication of the autotrophic growth potential of the strain. Olivieri et al. (2011) studied the autotrophic growth rate of *S. bacillaris* ACUF158 at pH 3.3, 6, and 8.3. In their study, *S. bacillaris* ACUF158 displayed a pH optimum of 6 with a *μ* of 0.79 day^−1^, while at pH 3.3 it was reduced to 0.33 day^−1^. In our experiments, *S. bacillaris* was cultivated at pH similar to the lowest pH tested by Olivieri et al. (2011) and we observed a similar growth rate.

In the only previous study on *C. acidophila* SAG 2045 (Gerloff-Elias et al., 2005), the authors reported two autotrophic pH optimums at pH 3 and 5 with maximum specific growth rate of 0.8 day^−1^. Gerloff-Elias et al. (2005) compared *C. acidophila* SAG 2045 at pH 3 and 5 to the neutrophilic *Chlamydomonas reinhardtii* SAG 11-32b grown at neutral pH reporting similar growth rate. The higher dark respiration rates found in *C. acidophila* were compensated by higher photosynthetic rates.

The poorest autotrophic performance in our experiment was observed from *C. pitschmannii* ACUF238. The only previous study on *C. pitschmannii* ACUF238 regards the eco-physiological characterization and isolation of the strain (Pollio et al., 2005). Growth optimum at pH 2.5 and temperature 37°C were found, while in our study the strain failed to grow under such conditions. Large intraspecific variations in key ecophysiological traits are common within natural populations. The *C. pitschmannii* strains were isolated by serial dilution from the site of Pisciarelli in mid-2003 and were immediately used for the experiments reported in Pollio et al. (2005). During long-term maintenance in the following 18 years, the strains have been treated against bacteria and fungi contamination and re-isolated. The combination of high nutrient concentrations provided by the culture media and the stability of light and temperature conditions (60 μmol m^−2^ s^−1^ and 24 ± 1°C, respectively) could have led to a laboratory selection of neutrophilic isolates with a temperature optimum closer to 25°C possibly explaining the disparities between the studies.

Another important observation in our work is that two acid tolerant species, *C. acidophila* SAG 2045 and *S. bacillaris* ACUF158, were able to grow mixotrophically with glucose displaying a higher growth rate than the corresponding autotrophic culture. In a previous study by Cuaresma et al. (2011), *C. acidophila* was cultivated mixotrophically without CO_2_ addition using acetic acid, glycerol, glucose, glycine, or starch as the sole carbon source. *Chlamydomonas acidophila* was able to utilize the tested organic substrates, except for acetic acid, but the growth rate and final biomass produced were significantly lower than, or in the case of glucose, similar, to the autotrophic control. A different strain used in our study may explain the variance between our results and Cuaresma et al. (2011). Our recent studies demonstrated that mixotrophy is a promising strategy to even double the biomass productivity (Abiusi et al., 2020b, 2021). Mixotrophic production of microalgae is already used at industrial scale to decrease microalgal production costs (Iwamoto, 2004; Ganuza and Tonkovich, 2016). We believe that mixotrophic cultivation of acid tolerant and acidophilic microalgae, by reducing the risk of contamination, will further facilitate this cultivation strategy.

We further studied glucose assimilation cultivating *C. acidophila* SAG 2045 under strictly heterotrophic conditions (no light) at 25°C. The heterotrophic specific growth rate (*μ*), we observed in this study (0.52 day^−1^) was comparable with the autotrophic *μ* of 0.72 day^−1^ observed by Gerloff-Elias et al. (2006) at 25°C. In the same study, the temperature optimum of *C. acidophila* SAG 2045 was reported to be 20°C with a drop of 25% in *μ* at 25°C. Suboptimal temperature might also explain the low biomass yield on substrate (*Y_x/s_*) (0.39 g_x_ g_s_^−1^) found in our study. In fact, a *Y_x/s_* of 0.7 g_x_ g_s_^−1^ has been previously reported in other *Chlymidomonas* species (Chen and Johns, 1996). Biomass yield on glucose is known to decrease as soon as the temperature optimum is exceeded, as also confirmed by the two *Galdieria* strains (Figure 4).

Glucose also increased the growth rate of *S. bacillaris* ACUF158 under mixotrophic conditions. Martinez et al. (1987) isolated a *S. bacillaris* from a sugar factory and reported that the mixotrophic growth rate was about 70% higher than autotrophic growth rate. Moreover, the same authors indicated that, when cultivated at neutral pH *S. bacillaris* could use sucrose, fructose, citrate, and acetate as carbon sources. At pH 3 in our mixotrophic experiments, acetic acid was lethal to all tested species. This was expected since acetic acid has a pKa of 4.76, so in our medium at pH 3 it was mainly present in the protonated form. Acetic acid can therefore enter the cell through passive diffusion. Once inside the cell, where the pH is close to neutrality, the undissociated acetic acid causes intracellular acidification. To counteract this acidification, protons have to be pumped out of the cells dissipating the proton motive force across the plasma membrane (Guldfeldt and Arneborg, 1998).

The most studied acidophilic microalgae species is *G. sulphuraria* with a growing research interest observed in the last decade (Sydney et al., 2019). *Galdieria sulphuraria* has been proposed as a novel source of protein (Abiusi et al., 2022), dietary fibers (Graziani et al., 2013), antioxidants (Carfagna et al., 2016) and of the blue pigment C-phycocyanin (Wan et al., 2016). For a long time, *G. sulphuraria* has been considered extremely photosensitive with light inhibition occurring at intensities above 200 μmol m^−2^ s^−1^ (Brock, 1978; Sloth et al., 2006) recently it was demonstrated (Abiusi et al., 2021); however, that *G. sulphuraria* ACUF064 can be cultivated at high light intensity by optimizing the specific light supply rate. This was done by optimizing the biomass concentration in the reactor (Abiusi et al., 2021). In the present work. we cultivated two *G. sulphuraria* strains at 100 and 300 μmol m^−2^ s^−1^ and started the experiment at biomass concentrations of 0.9 g L^−1^ and 1.5 g L^−1^, respectively (Supplementary Figures 5, 6). At these biomass concentrations, the specific light supply rate was below 9.5 μmol_ph_ g_x_^−1^ s^−1^, the upper limit previously reported for photoinhibition (Abiusi et al., 2021). Absence of photoinhibition was confirmed by the photosystem II maximum quantum yield (*QY*, Fv/Fm) which was in a higher range reported for *G. sulphuraria* (Abiusi et al., 2021; Uwizeye et al., 2021). Moreover, linear growth was observed from the first day (Supplementary Figures 5, 6) further confirming the adaptation of those strains for both the light intensities applied. These results obtained with two strains, confirm that *G. sulphuraria* can successfully be cultivated autotrophically at a high light intensity with a good biomass productivity.

Graziani et al. (2013) performed the largest screening ever performed on *G. sulphuraria*, comparing autotrophic and heterotrophic specific growth rates of 42 strains belonging to ACUF. In the autotrophic screening, the *G. sulphuraria* strains were cultivated at pH 1.5, 36°C without CO_2_ addition and the medium contained only 40 mg L^−1^ of nitrogen, 14 times lower than the nitrogen concentration used in our work. Heterotrophic cultivation was conducted using 3% glycerol as the source of organic carbon. *Galdieria sulphuraria* ACUF064 was the best performing strain under both autotrophic and heterotrophic conditions. *Galdieria sulphuraria* ACUF064 had a similar autotrophic *μ* as *G. sulphuraria* ACUF074 but under heterotrophic conditions, the *μ* of *G. sulphuraria* ACUF064 was the double of *G. sulphuraria* ACUF074, 1.0 day^−1^ and 0.5 day^−1,^ respectively. The difference in heterotrophic *μ* between our study and Graziani et al. (2013), may be explained by different growing conditions (e.g., medium composition, organic substrate).

The lack of standardized procedures in the screening of acid tolerant and acidophilic microalgae was the main cause of the discrepancies between our findings and the previous studies. Moreover, previous studies mainly measured the specific growth rate (*μ*), which is not sufficient to predict bulk growth at biomass concentrations used in large scale production. Commercial production of microalgae is better characterized by linear growth, such as volumetric (g L^−1^ day^−1^) or areal (g m^−2^ day^−1^) productivity. Van Wagenen et al. (2014) proposed a microplate-based method for high-throughput screening of microalgae, based on the measurement of two input parameters: specific growth rate as a function of light intensity and light absorbance coefficient. Using these two inputs it was possible to predict the volumetric biomass productivity. This type of a high-throughput screening procedures can offer a solution to obtain industrially relevant information on algal strains. Despite hundreds of microalgal strains able to grow at pH below 3 having been isolated (D’Elia et al., 2018) and kept at culture collections, information on the biomass productivities of these strains are limited to a few. Standardized high-throughput screenings are required to exploit the potential of unexplored groups of microalgae.

### Future Outlook

In this study, we demonstrated that microalgae cultivated at pH below 3 can have biomass productivities comparable to neutrophilic strains. In this section, we will discuss possible advantages and biotechnological applications of acid tolerant and acidophilic microalgae.

Biomass productivity of an illuminated algal culture can be doubled by utilizing a mixotrophic cultivation strategy (Abiusi et al., 2020a, 2022). In the current study, two acid tolerant strains, *C. acidophila* SAG 2045 and *S. bacillaris* ACUF158, demonstrated an increased growth rate when cultivated in the presence of glucose, while *G. sulphuraria* is known to be able to grow mixotrophically on glucose and glycerol (Sloth et al., 2006). Mixotrophic cultivation of those acid tolerant and acidophilic strains is expected to further increase biomass productivity making them more productive than most of the neutrophilic microalgal strains cultivated autotrophically. When neutrophilic microalgae are cultivated in presence of organic substrate, contamination by heterotrophic bacteria and fungi is a considerable challenge (Unnithan et al., 2014). Contaminating microbes have a growth rate faster than microalgae and can therefore outcompete algae for organic carbon utilization. Cultivating microalgae at low pH can be a worthwhile strategy to prevent contaminants. Previous works indicated that *G. sulphuraria* can be cultivated axenically in a lab scale mixotrophic reactor for over a month (Abiusi et al., 2021, 2022). Another work reports that at pH 2 *G. sulphuraria* was the main organism growing in unsterilized primary municipal waste water (Henkanatte-Gedera et al., 2017). Moreover, the low pH reduced the initial bacterial population by 98% and removed all the pathogens originally present in the waste water. Therefore, cultivation of microalgae at low pH might offer a means to bioremediate urban waste water (di Cicco et al., 2021) or valorize agro-industrial side streams (Scherhag and Ackermann, 2020), while minimizing the risk of contaminations by unwanted microorganisms.

Acid tolerant and acidophilic microalgae are often found in mining effluents characterized by low pH and high concentration of heavy metals (Dean et al., 2019). One exciting application is the use of those microalgae in rare earth metal biorecovery (Minoda et al., 2015). Numerous metals, including rare earth elements, can be readily dissolved in aqueous acid and selectively bioaccumulated in acidophilic microalgae from which they are then recovered.

Extremophilic organisms have also received increasing attention for the production of metabolites and enzymes that are commercially relevant for chemical, pharmaceutical, and food industries. Extremophiles have already been used for the production of extremozymes able to catalyze chemical reactions at an unusually high or low pH, temperature, or pressure. Such enzymes broaden the operational range of bioprocesses. Recently, the presence glucoamylases active at pH 2–2.5 and 80°C have been demonstrated in *G. sulphuraria* (Rahman et al., 2020). In the whole genome analysis of *G. sulphuraria*, a range of genes with high similarity with other extremozymes have been identified (Shrestha and Weber, 2007), which gives an indication that extremophilic microalgae can be used as a source extremozymes.

Extremophiles can also be used to produce extremolytes (Malavasi et al., 2020), small organic molecules, which allow extremophiles to withstand their extreme environments. They might have bioactive properties usable for medical purposes and in food industry (Becker and Wittmann, 2020). Floridoside is a glycoside accumulated by almost all red algae, including *G. sulphuraria,* under a high osmotic pressure (Martinez-Garcia and van der Maarel, 2016). This compound has been proposed for preventing biofouling in aquaculture (Callow and Callow, 2002), as a potential therapeutic agent to modulate immune response (Kim et al., 2013) and to promote bone formation (Ryu et al., 2015). The development of industrial applications for floridoside is hindered by low availability. *Galdieria sulphuraria* is demonstrated to accumulate high concentration of this compound and is a promising organism for industrial production of floridoside (Martinez-Garcia and van der Maarel, 2016).

Finally, acidophilic microalgae have been envisaged as novel source of pigments such as lutein (Cuaresma et al., 2011) and C-phycocyanin (Abiusi et al., 2022). C-phycocyanin extracted from several members of the cyanidiales family, known acid and thermophilic organisms, have reportedly exceptional thermo- and acid stability (Rahman et al., 2017; Ferraro et al., 2020a). The higher stability compared to the C-phycocyanin of other microalgae (e.g., *Spirulina*) is due to a residue mutation on the outside of the conserved regions (Ferraro et al., 2020b). Those traits open the opportunity to use said pigments in commonly pasteurized sparkling beverages, characterized by a low pH.

Acid tolerant and acidophilic microalgae have applications in several industrial processes such as the ones listed in this section. However, most of the current knowledge on possible new products and applications comes almost solely from studies conducted on *G. sulphuraria*. The combination of high throughput screening and omics techniques can be employed to select new productive strains as source of high value compounds.

## Conclusion

In the present study, six microalgal strains were cultivated at a pH below 3. Utilizing acid growth conditions is a strategy to prevent unwanted microbial contaminations in autotrophic and specifically mixotrophic microalgae cultivation. Five of the tested strains showed an autotrophic biomass productivity comparable with other commercially relevant neutrophilic microalgae, indicating the potential of these microalgae for autotrophic biomass production. Two strains were also able to grow mixotrophically on glucose displaying higher growth rates than the corresponding autotrophic cultures. The ability to grow heterotrophically on glucose was tested on three strains. All of the strains grown heterotrophically lost their pigmentation in the darkness and displayed a specific growth rate between 0.5 and 1 day^−1^, comparable to other commercially relevant microalgal species. Due to the reduced risk of microbial contaminations and biomass productivity comparable to neutrophilic microalgae, acid tolerant, and acidophilic microalgae are promising candidates for mass cultivation.

## Data Availability Statement

The raw data supporting the conclusions of this article will be made available by the authors, without undue reservation.

## Author Contributions

FA designed and conducted experiments, analyzed and interpretated the data, and drafted the manuscript. ET conducted a part of the experiments and analyzed the corresponding data. AP provided the strains and assisted with interpretation of the data. RW obtained funding and assisted with interpretation of the data. MJ contributed to the design of the experiments, interpreted data, and drafted the manuscript. All authors contributed to the article and approved the submitted version.

## Funding

This work was supported by AlgaePARC, Wageningen University.

## Conflict of Interest

The authors declare that the research was conducted in the absence of any commercial or financial relationships that could be construed as a potential conflict of interest.

## Publisher’s Note

All claims expressed in this article are solely those of the authors and do not necessarily represent those of their affiliated organizations, or those of the publisher, the editors and the reviewers. Any product that may be evaluated in this article, or claim that may be made by its manufacturer, is not guaranteed or endorsed by the publisher.
