# Supplementary material for: Acid Tolerant and Acidophilic Microalgae: An Underexplored World of Biotechnological Opportunities
Source: Front Microbiol. 2022 Jan 27;13:820907. doi: 10.3389/fmicb.2022.820907 (PMC8829295; doi:10.3389/fmicb.2022.820907)
Supplement: Supplementary file 1 [file Data_Sheet_1.docx]

**Supplementary figures**

**Supplementary Figure S1.** Autotrophic growth of *Chlorella sorokiniana* SAG 211-8k cultivated at pH 6.8±0.1, 37°C and at two light intensities: 300 µmol·m^-2^·s^-1^ (orange triangles) and 100 µmol·m^-2^·s^-1^ (blue dots).

**Supplementary Figure S2.** Autotrophic growth of *Stichococcus bacillaris* ACUF158 cultivated at pH 2.9±0.3, 27°C and at two light intensities: 300 µmol·m^-2^·s^-1^ (orange triangles) and 100 µmol·m^-2^·s^-1^ (blue dots).

**Supplementary Figure S3.** Autotrophic growth of *Chlamydomonas acidophila* SAG 2045 cultivated at pH 2.2±0.1, 27°C and at two light intensities: 300 µmol·m^-2^·s^-1^ (orange triangles) and 100 µmol·m^-2^·s^-1^ (blue dots).

**Supplementary Figure S4.** Autotrophic growth of *Viridiella fridericiana* ACUF035 cultivated at pH 2.1±0.2, 27°C and at two light intensities: 300 µmol·m^-2^·s^-1^ (orange triangles) and 100 µmol·m^-2^·s^-1^ (blue dots).

**Supplementary Figure S5.** Autotrophic growth of *Galdieria sulphuraria* ACUF074 cultivated at pH 2.0±0.2, 37°C and at two light intensities: 300 µmol·m^-2^·s^-1^ (orange triangles) and 100 µmol·m^-2^·s^-1^ (blue dots).

**Supplementary Figure S6.** Autotrophic growth of *Galdieria sulphuraria* ACUF064 cultivated at pH 1.9±0.2, 37°C and at two light intensities: 300 µmol·m^-2^·s^-1^ (orange triangles) and 100 µmol·m^-2^·s^-1^ (blue dots).

**Supplementary Figure S7.** Autotrophic growth of *Chlamydomonas pitschmannii* ACUF238 cultivated at pH 2.8±0.2, 27°C and 100 µmol·m^-2^·s^-1^ (blue dots).


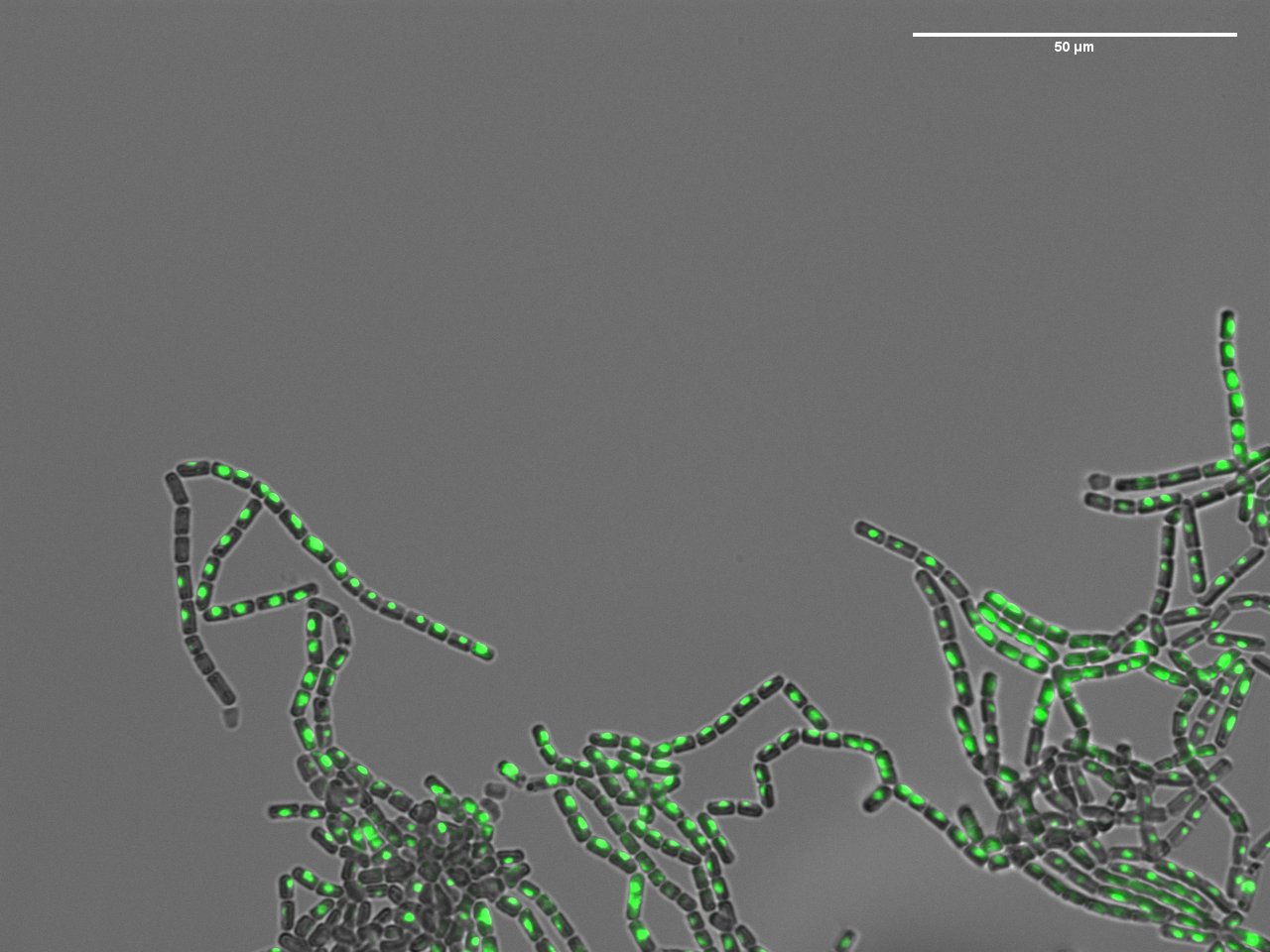


**Supplementary Figure S8.** Fluorescent micrograph of a sample of *Stichococcus bacillaris* ACUF158 with SYBER Green I DNA-stain taken at the end of the mixotrophic experiment.

**
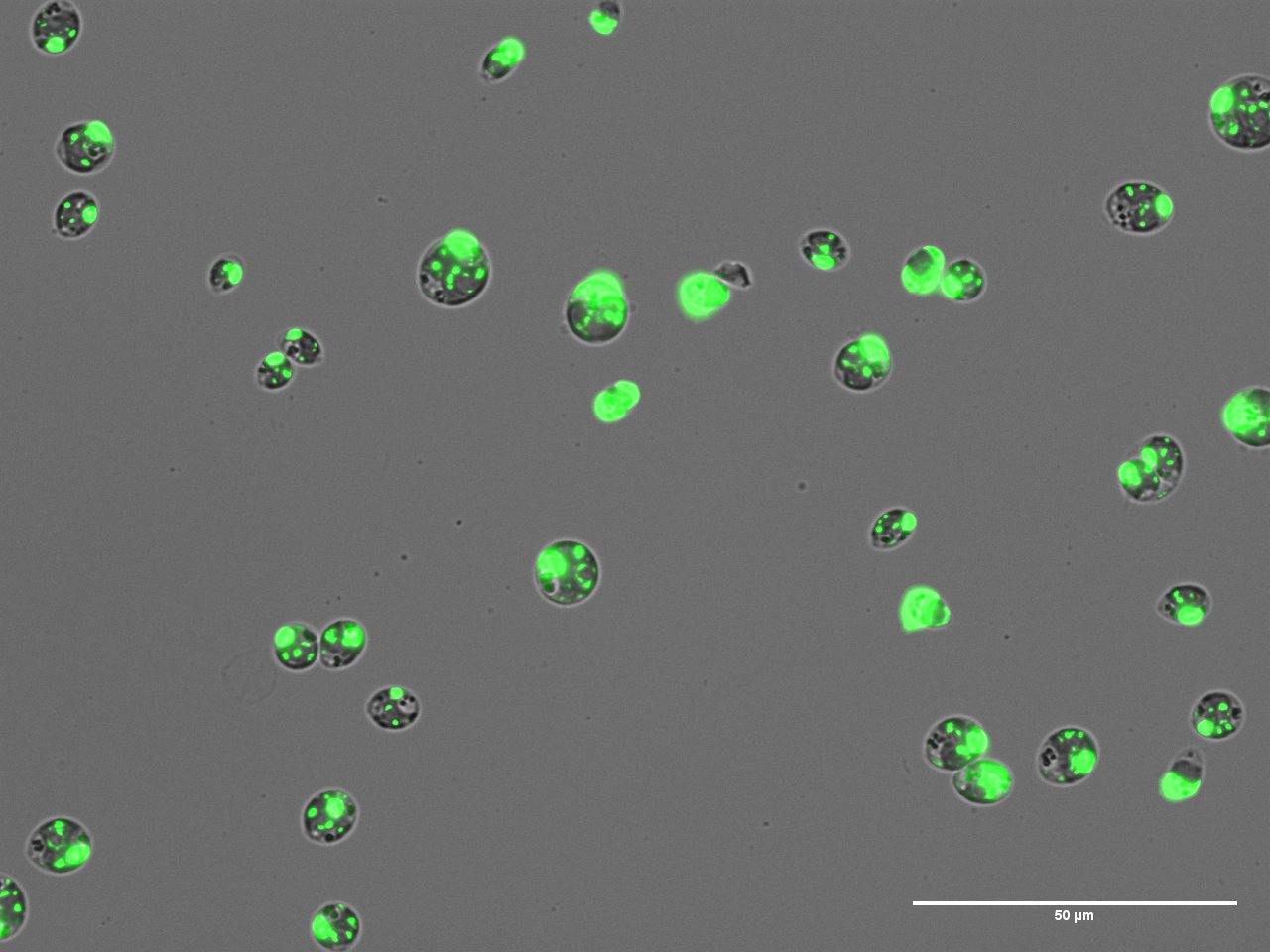
**

**Supplementary Figure S9.** Fluorescent micrograph of a sample of *Chlamydomonas acidophila* SAG 2045 with SYBER Green I DNA-stain taken at the end of the mixotrophic experiment.

**
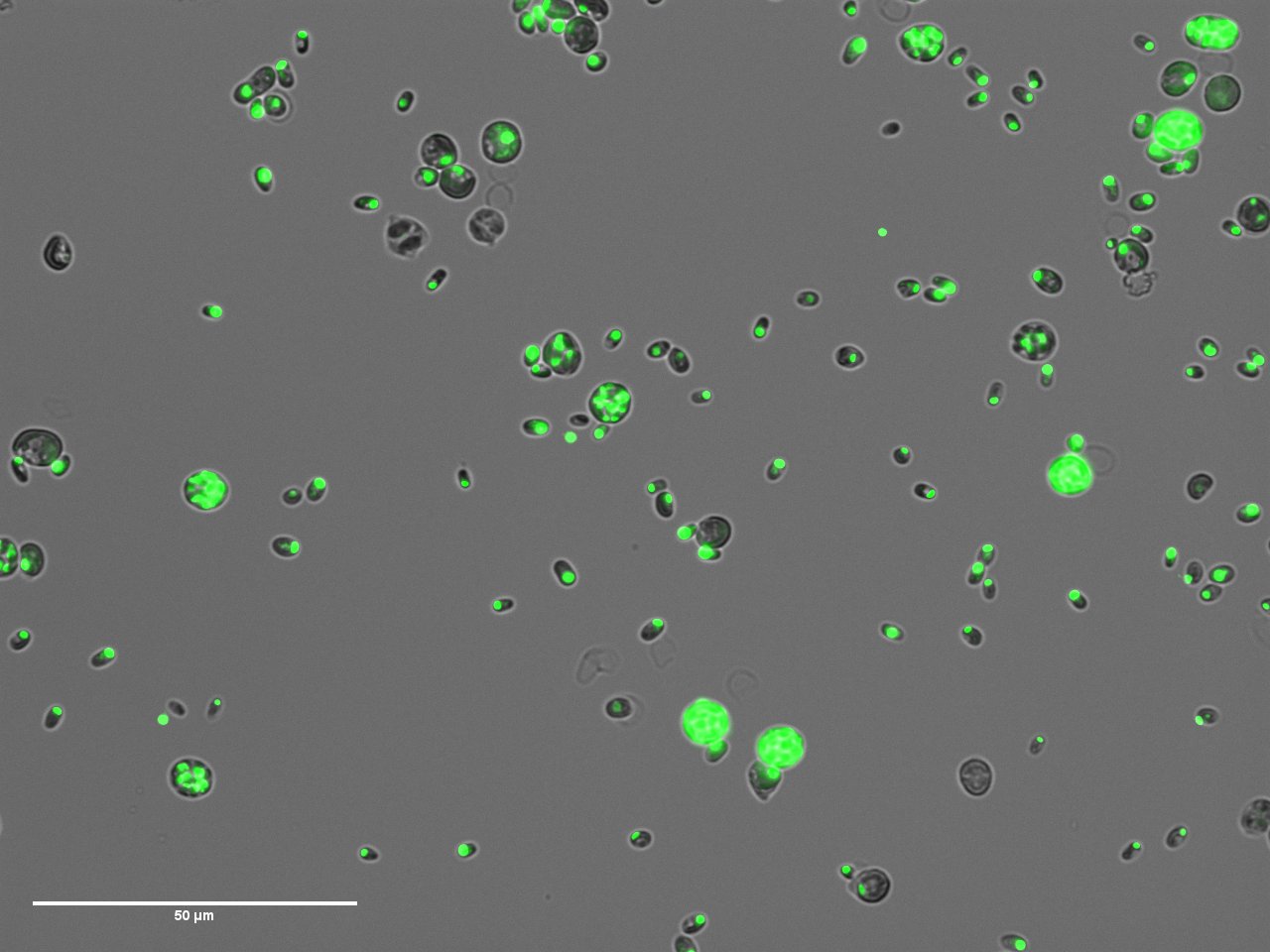
**

**Supplementary Figure S10.** Fluorescent micrograph of a sample of *Viridiella fridericiana* ACUF035 with SYBER Green I DNA-stain taken at the end of the mixotrophic experiment.
